# Modelling the risk of professional disengagement from a cohort study of 181,676 workers in the south of France

**DOI:** 10.1371/journal.pone.0346149

**Published:** 2026-03-31

**Authors:** Gauthier Bellagamba, Vincent Houtin, Anne Landi, Marie-Pascale Lehucher-Michel

**Affiliations:** 1 Groupement Interprofessionnel Médico-Social (GIMS), Marseille, France; 2 Aix-Marseille Univ, CEReSS, Marseille, France,; 3 APHM, Groupe Hospitalier Timone, CRPPE, Marseille, France; Gazi University, Faculty of Health Sciences, TÜRKIYE

## Abstract

**Objective:**

This study determines a set of medical, occupational, and social risk factors of professional disengagement that can be documented early in occupational health practice.

**Method:**

This study was carried out on a cohort of French workers. Data were extracted from computerised occupational health medical follow up records. The professional disengagement risk was assessed by healthcare professionals. Logistic regression models were applied.

**Results:**

181,676 workers were included. Of these, 4.05% had a high professional disengagement risk index (PDRI) and 13.0% a moderate risk. Women (OR: 1.21) and 50–61-year-old (OR: 5.17) workers appear to be the most at high risk. White-collar (OR: 1.63) and blue-collar (OR: 1.60) have a higher at risk compared with manager. The main disorder risk factors of high PDRI were mental disorders (OR: 8.79), musculoskeletal disorders including shoulder (OR: 6.23), arm (OR: 3.80), or hand (OR: 3.21) and kidney disorders (OR: 5.57). Recognition of a professional disengagement (OR: 7.55) or long/iterative sick leave (OR: 5.08) is associated with high PDRI. Occupational exposure associated with high PDRI include “poor interpersonal relationships” (OR: 6.05), “job insecurity” (OR: 4.14) and “conflicts of values” (OR: 3.88). Exposure to noise (OR: 1.33) and heavy work (OR: 1.25) are associated with a high PDRI, however some physical exposures appear to be ‘protective’ such as lower limb postures (OR: 0.68) or whole-body vibrations (OR: 0.64).

**Conclusions:**

Mental and musculoskeletal disorders, poor working relationships, job insecurity and conflicts of values all appear to be avoidable risk factors for professional disengagement These results reinforce the importance of targeted prevention and orientates future research to refine the accuracy of the variables and better distinguish gradients and thresholds of risk.

**What is already known:**

Numerous studies have investigated the factors facilitating or limiting a return to work after professional disengagement.

**What this study adds:**

This study highlights the medical and professional factors which should be early documented to prevent professional disengagement.

## Introduction

Professional disengagement (PD) is a multifactorial process, often long and latent, which results in the lasting exclusion of a worker from employment [1,2]. Its high human and socioeconomic cost leads us to consider the prevention of PD and job retention as public health priorities [1].

A worker’s PD can be broadly described in three stages. The earliest stage corresponds to the occurrence of one or more risk factors with no impact on the worker’s ability to work, and the second stage corresponds to discomfort at work that may be associated with medically prescribed sick leave. The final stage, in which the worker’s state of health is incompatible with their job, may lead to dismissal.

For the last two stages, a great deal of work has been done. Some studies have examined the factors limiting return to work after the onset of a disease (breast cancer, myocardial infarction, depression, stroke, etc.) [3–8]. Others, focusing on the causes of medical unfitness, have shown that mental and musculoskeletal illnesses are the main risk factors [9].

For the earliest stage, there is less data available, and what there is mainly concerns workers’ perceptions of their work ability, measured using a self-administered questionnaire, the Work Ability Index (WAI). It has been identified that age (62 and over), mental disorders, and musculoskeletal disorders of the neck and back are risk factors for reduced work ability [10].

However, this measure of the worker’s perception, an essential component in the PD process, remains subjective and fragmented.

A Professional Disengagement Risk Index (PDRI) classifying employees into four increasing levels according to their risk (0 = zero, 1 = low, 2 = medium, 3 = high) of PD has recently been established for use by healthcare professionals monitoring workers [10]. This index has the advantage of taking into account medical factors, working conditions, workers’ perceptions of their work capacity, and the impact on their work capacity of risk factors identified by healthcare professionals. These risk factors can be objectively assessed for each worker using computerized occupational health service data entered by healthcare professionals.

In order to help prevent the risk of PD as early as possible, it is important to be able to identify the risk factors associated with a PDRI estimated by the healthcare professional to be at a medium (PDRI 2) to high (PDRI 3) level.

In this context, the aim of this study is to use routine data from an occupational health service (OHS) to identify risk factors associated with PDRI 2 or 3.

## Method

### Type of study and population

This observational study was based on data from an OHS cohort in the south of France where the OHS actively follows up to nearly 182,000 employees working for companies in the secondary and tertiary sectors.

The employees included in the study were those who had received at least one hiring, periodic or occasional check-up between 01.01.2022 and 31.12.2024.

### Data sources and variables of interest

All pseudonymised data were extracted directly from computerised occupational health records, the 06.01.2025.

The socio-professional data extracted were gender, age, seniority in post, professional category, type of employment contract, and sector of activity.

Professional disengagement Risk Index (PDRI).

The score used in this study to assess PDR was the PDRI (Supplemental S1 Table).

The PDRI rating was schematically based on a double-entry table comprising the employee’s clinical data and an assessment of his or her well-being at work [11,12]. For the clinical part, if there is a known risk factor that could affect life expectancy or a history that could be reactivated, the worker will be classified as PDRI 1. When a pathology is present, a clinical sign is detected or the worker reports a complaint, the latter will be classified as PDRI 2. Finally, if the employee is classified as PDRI 2 and there is also discomfort at work, they will be classified as PDRI 3. For the well-being at work section, a well-being at work score of < 7/10 reported by the worker will classify them as PDRI 1. If there is an impact on health or if the worker no longer sees themselves staying with the company in the long term, they will be PDRI 2. Finally, if both of these last two elements are present, they will be classified as PDRI 3.

When all these elements were known by the health professional, they could estimate a PDRI ranging from 0 to 3 (zero-low-medium-high risk).

To assess the validity of the PDRI, a study has assessed the correlation between the PDRI and the work ability index (WAI). The high correlation suggests that the PDRI appears to be a valid tool for the early detection of the risk of PD. Thus, a high risk of PD identified by the PDRI was correlated with a decrease in work ability self-assessed using the WAI [12].

Employees with PDRI levels 2 and 3 were considered to be vulnerable.

The study focused on PDR, which occurs when an individual has at least one pathology and this pathology interferes with his or her work. It did not study cases of PD due to non-medical causes or unrelated to working conditions.

#### Clinical data.

Disorders and medical histories were coded using the ICD10 and ICD11 classification [13]. The variables analysed were those recognized as potentially causing discomfort at work.

We decided to group them into 19 categories according to the type of disorder or their location: Musculoskeletal Disorder (MSD) arm/elbow, MSD shoulder, MSD hand/wrist, MSD cervical, MSD back, MSD lower limbs, Secondary MSD, Mental health disorders, Cancers, Cardiovascular disorders, Hypertension, Neurological disorders, Vision disorders, Respiratory disorders, Sleep disorders, Diabetes, Hypercholesterolemia, Kidney disorders, Obesity (Supplemental S2 Tables).

#### Professional constraints and exposures.

Professional constraints and exposures were coded by occupational health professional using the Anses thesaurus. [14] The codes of this thesaurus were exploited at the most detailed level available (5 possible levels).

Organisational constraints [15] were grouped into 9 categories: Remote work, Workload variability or unpredictable, Working under time constraints, Frequent night shift, Solitary work, Shift work, Non-standard hours, Business trip, Shared workspace, Regular contact with the public. [14] (Supplemental S2 Tables)

Harmful biomechanical exposures were grouped into 10 categories: Heavy work, Repetitive upper limb movement, Repetitive spinal movement, Repetitive lower limb movements, Non-localised repetitive movements, Upper limb posture, Spinal posture, Lower limb posture, Whole body posture, Non-localised posture [14]. Non-localised repetitive motion/ posture refers to multi-segment movements/postures. (Supplemental S2 Tables)

Physical exposures at the workplace were grouped into 12 categories: Extreme temperatures, Whole body vibration, hand-arm vibration, Noise, Ionizing radiation, Bad weather, Electricity, Fires & explosions, Risk of falls at the same-level, Risk of falls from height, Professional road risks, Computer work. [14] (Supplemental S2 Tables)

The Psycho-Social Risk (PSR) Factors were extracted by combining queries in the exposure thesaurus [14] and in the Well-Being at Work evaluation module: Emotional demands, Lack of autonomy, Poor interpersonal relationships, Values conflicts, Workload, Job insecurity, Violence. (Supplemental S2 Tables)

In our database, the variable “chemical exposure” indicates whether an employee is exposed to at least one chemical product in the workplace.

Well-Being at Work was assessed using a visual scale ranging from 0 to 10. A score of less than 7 indicates that well-being at work is impaired [16,17].

To date, healthcare professionals do not indicate the date of occurrence of each independent event. Thus, data for each independent event have been included only if their documentation date is prior to or equal to the date of the last PDRI.

#### Administrative situation.

The administrative situation of the employees was also examined.

In France, an employee may be recognized as a Disabled Worker (DW). This is an administrative decision that entitles the employee to a range of measures to help them remain in work or find a job. [18]

A worker may be entitled to a disability allowance. This compensates for loss of earnings when an illness or accident of non-occupational origin results in a reduction of at least two-thirds in working or earning capacity. [19] When this reduction in working capacity is work related (following an accident at work or a work-related illness), it is referred to as temporary or permanent incapacity. [20]

Temporary or permanent adaptation of the workstation, a measure recommended by the occupational physician, was also entered.

For long-term conditions whose seriousness and/or chronic nature require prolonged treatment, the ability to work was not considered.

Long and/or iterative work stoppages refer to work stoppages lasting more than 60 days and/or the existence of more than 2 work stoppages over a 12-month period.

#### Habits and lifestyle.

Habits and lifestyle were filled in at each visit, on a declarative basis by the employee, and then assessed by the health care professional.

### Occupational health monitoring

In France, all employees benefit from occupational medical surveillance carried out by an OHS, which includes a series of visits to a health professional (occupational physician or nurse). A distinction is made between hiring visits (or pre-employment medical examination), periodic visits (or routine occupational health consultations), and occasional visits (or unscheduled occupational health consultations).

The frequency of periodic visits is determined by the occupational physician for a period not exceeding five years.

Occasional visits include pre-reinstatement and resumption visits (after sick leave or maternity leave) or visits at the request of the employee, the employer or a physician.

### Bias management

*Selection bias.* Workers were included as soon as they were hired. This practice ensures that all workers are systematically monitored. The only significant selection bias concerns workers on short-term fixed-term contracts who do not have time to receive an invitation to attend a visit and those who do not attend the visit despite being invited. The former represent around 10% of total employment [21] and the others 25% of the working population. It should be noted that workers who do not turn up for recruitment or periodic visits can systematically benefit from an occasional visit in the event of a problem.

*Measurement and classification bias*. Depending on the worker’s situation, the documentation of certain ‘secondary’ clinical or occupational factors could lead to an under-evaluation of these factors. Nevertheless, there was no reason for this bias to be differential. Furthermore, for greater homogeneity, the documentation of the variables followed the methodological practices set out above (PDRI, ICD, Anses thesaurus), limiting the risk of classification bias.

### Statistical analysis

*Missing data.* For independent variables with missing values, if more than 90% of the independence test results were non-significant, the data were considered missing completely at random [22]. Thus, missing values were imputed using a MICE multiple imputation algorithm. Variables whose missing data distributions suggested that the data were missing non-randomly were excluded.

In the context of the study, the clinical visit protocols imply that there are no missing data concerning clinical, professional constraints, exposures and administrative status data. The absence of coding qualifies the absence of the event. Independence tests were conducted between variables with missing data and the variables gender, age group and sector of activity.

*Regression model.* A polytomous logistic regression model was used to study the association between the PDRI levels (PDRI0 VS PDRI1, PDRI2, PDRI3), and the independent variables. Only the variables whose data could not be imputed were not included in the model.

Penalization parameters were added to the logistic regression model. ElasticNet penalized regression (L1 + L2 penalization) aims to reduce the risk of overfitting, particularly related to correlations between variables. The model was penalized iteratively by increasing the regularization parameter λ by a step of λn + 1 = 10*λn. The variation in performance was measured at each iteration. If performance changes over the iterations and the L2 norm reduction value increases, then the penalty highlights a high probability of multicollinearity. If the performance (AUC) is stable, this indicates that potential collinearities have no influence.

Performance measurement was used to decide which regression model—simple, weighted, or penalized—was most appropriate.

A hierarchical classification model was tested. This model groups individuals according to their similarity (e.g., professional category, sector of activity) highlighting hierarchical relationships between classes and thus correcting for biases related to correlations between observations (intra-group correlation) [23].

The clinical data included in the PDRI were also considered as independent variables in the regression model. Undersampling techniques were used to test the balance of the class distribution.

*Performance measures*. The model’s performance was evaluated using the Area Under the Curve (AUC). The AUC corresponds to the area under the ROC (Receiver Operating Characteristic) curve, which represents the trade-off between sensitivity (true positive rate) and specificity (1 – false positive rate) for all possible decision thresholds.

The Intraclass Correlation Coefficient (ICC) was used to decide whether to retain the hierarchical model. The ICC measures the proportion of variance in a variable attributable to differences between groups relative to the total variance. The higher the ICC, the more appropriate it is to take the hierarchical structure into account. If the ICC is low, the observations have no hierarchical relationship.

An assessment of the risk of model overfitting was performed using the k-fold cross-validation method. This method allows the robustness of the regression model to be estimated without the need for an independent test sample. The overall sample was divided into five sub-samples of equal size. Comparing the performance of the model applied to each subsample made it possible to assess the stability of the model. Low variability in the odds ratios (OR) between the subsamples indicates a low risk of overfitting, and stable performance between the models indicates good model robustness.

In order to evaluate the model’s discriminatory power on samples smaller than that of the study, the five subsamples were reduced to 20% of the overall sample.

All statistical analyses were carried out using Python packages: statsmodels, sklearn.

### Ethics

This processing operation has been declared compliant with the reference methodology MR-004 that governs the processing of personal data for the purposes of study, evaluation or research not involving the human person (CNIL reference no. CNIL2233048). This study does not involve human participants.

## Results

A total of 181,676 employees were included in the study. The PDRI levels were distributed as follows: 62.9% PDRI 0 (no risk), 20.1% PDRI 1 (low risk), 13.0% PDRI 2 (medium risk), and 4.05% PDRI 3 (high risk).

Table 1 show the results of the univariate analysis applied to PDRI levels according to medico-socio-professional characteristics. In the socio-professional groups, there is an over-representation of managers with low vulnerability (PDRI0: 15.1%) VS high vulnerability (PDRI3: 7.47%), unlike white-collar workers for whom the trend is reversed (low vulnerability: 42.7% VS high vulnerability: 54.9%). For the different age groups, the results show an over-representation of high vulnerability among the oldest age categories. In terms of professional activity, there is an over-representation of vulnerable employees (29.9% of PDRI3 VS 16.9% of PDRI0) in the health and social work sectors, unlike the information and communication sector (2.04% of PDRI3 VS 4.65% of PDRI0).

**Table 1:** PDRI levels according to socio-professional characteristics and administrative situations.

n (%)	PDRI levels			
**PDRI 0**	**PDRI 1**	**PDRI 2**	**PDRI 3**
Sample	114206 (62.9)	36475 (20.1)	23634 (13.0)	7361 (4.05)
Gender				
male	59169 (51.8)	17239 (47.3)	10575 (44.7)	2741 (37.2)
female	55037 (48.2)	19236 (52.7)	13059 (55.3)	4620 (62.8)
Socio-professional group (**GSP**)				
Craftsmen, shopkeepers and company directors	815 (0.71)	231 (0.63)	157 (0.66)	31 (0.42)
Managers	17291 (15.1)	4421 (12.1)	2666 (11.3)	550 (7.47)
Intermediate occupations	24637 (21.6)	8374 (23.0)	5412 (22.9)	1412 (19.2)
White-collar	48810 (42.7)	16489 (45.2)	11040 (46.7)	4038 (54.9)
Blue-collar	22653 (19.8)	6960 (19.1)	4359 (18.4)	1330 (18.1)
Current contract				
Open-ended contract	75950 (66.5)	27254 (74.7)	18501 (78.3)	6332 (86.0)
Fixed-term contract	16062 (14.1)	4112 (11.3)	2219 (9.39)	389 (5.28)
Temporary employment	9042 (7.92)	1422 (3.90)	695 (2.94)	62 (0.84)
Other	13152 (11.5)	3687 (10.1)	2219 (9.39)	578 (7.85)
Sector of activity				
Indus. manufacturing, extract. & other	6532 (5.72)	1932 (5.3)	1328 (5.62)	398 (5.41)
Commerce, transport, accommodation & catering	36104 (31.6)	11134 (30.5)	7041 (29.8)	2290 (31.1)
Information and communication	5316 (4.65)	1209 (3.31)	683 (2.89)	150 (2.04)
Finance and insurance	4807 (4.21)	1221 (3.35)	886 (3.75)	202 (2.74)
Real estate activities	1952 (1.71)	642 (1.76)	412 (1.74)	132 (1.79)
Specialised activities, admin. & business support	27094 (23.7)	7223 (19.8)	4459 (18.9)	1156 (15.7)
Public admin., education	7328 (6.42)	3601 (9.87)	2015 (8.53)	466 (6.33)
Health and social work activities	19339 (16.9)	7461 (20.5)	5587 (23.6)	2202 (29.9)
Other service activities	5734 (5.02)	2052 (5.63)	1223 (5.17)	365 (4.96)
Age category				
15 to 24 years old	16601 (14.5)	2778 (7.62)	1079 (4.57)	149 (2.02)
25 to 34 years old	37753 (33.1)	8369 (22.9)	3650 (15.4)	1049 (14.3)
35 to 49 years old	36883 (32.3)	12228 (33.5)	7508 (31.8)	2385 (32.4)
50 to 61 years old	18385 (16.1)	10202 (28.0)	8526 (36.1)	2760 (37.5)
62 or over	4584 (4.01)	2898 (7.95)	2871 (12.2)	1018 (13.8)
Seniority				
Employee seniority > 5 years	24166 (21.2)	12310 (33.8)	9605 (40.6)	3798 (51.6)

Habits and lifestyle were also studied in a univariate analysis. Among the categories in which high vulnerabilities were over-represented were ‘unbalanced’ nutrition (23.9% of PDRI3 VS 11.7% of PDRI0), not doing a physical activity (55.1% VS 37.2%), and regular sleep problems (42.9% VS 6.56%).

With regard to well-being at work, a tendency for low well-being is more frequent in the high vulnerability group (50.7%) than in the low vulnerability group (5.54%).

Finally, when a disorder was indicated during the visit, regardless of the equipment concerned or the location, the proportion of “vulnerable” employees appeared to be higher. Mental disorders (28.0% of PDRI3 VS 1.58% of PDRI0) were the most affected by this over-representation.

For the multivariate analysis, we compared PDRI levels 1, 2, and 3 were then compared with PDRI0 as a reference. The results of the simple logistic regression models are presented in Tables 2, 3, 4 (and Supplemental S1 Fig).

**Table 2:** Multivariate analysis of PDRI levels according to socio-professional characteristics and administrative situations.

	PDRI 1 (VS 0) OR	PDRI 2 (VS 0) OR	PDRI 3 (VS 0) OR
Gender (ref: man)			
woman	1.07 [1.04 - 1.10]	1.08 [1.04 - 1.12]	1.21 [1.13 - 1.29]
Age category (ref: 15–24 years old)			
25 to 34 years old	1.21 [1.15 - 1.27]	1.28 [1.19 - 1.38]	2.20 [1.84 - 2.64]
35 to 49 years old	1.48 [1.41 - 1.56]	1.98 [1.84 - 2.13]	3.46 [2.90 - 4.13]
50 to 61 years old	1.95 [1.85 - 2.06]	2.95 [2.73 - 3.19]	5.17 [4.31 - 6.18]
62 years old or more	2.01 [1.87 - 2.16]	3.31 [3.02 - 3.63]	6.93 [5.71 - 8.40]
Socio-professional group (ref: managers)			
Craftsmen, shopkeepers, company directors	1.17 [1.00 - 1.38]	1.26 [1.03 - 1.53]	1.35 [0.92 - 1.99]
White-collar	1.25 [1.19 - 1.30]	1.22 [1.15 - 1.29]	1.63 [1.46 - 1.82]
Intermediate occupations	1.20 [1.15 - 1.26]	1.19 [1.12 - 1.27]	1.18 [1.05 - 1.32]
Blue-collar	1.33 [1.26 - 1.41]	1.19 [1.11 - 1.28]	1.60 [1.41 - 1.83]
Current type of contract (ref: other)			
Fixed-term contract	0.92 [0.87 - 0.97]	0.83 [0.77 - 0.89]	0.61 [0.53 - 0.71]
Open-ended contract	1.02 [0.98 - 1.07]	0.95 [0.90 - 1.01]	1.17 [1.05 - 1.29]
Temporary employment	0.57 [0.52 - 0.62]	0.46 [0.41 - 0.52]	0.21 [0.15 - 0.27]
Business sector (ref: Indus manufacturing, extract.)			
Commerce, transport, accommodation & catering	1.04 [0.98 - 1.10]	0.93 [0.87 - 1.01]	0.89 [0.78 - 1.01]
Finance, insurance	0.89 [0.81 - 0.97]	0.83 [0.74 - 0.93]	0.65 [0.53 - 0.79]
Health, social work	1.03 [0.97 - 1.10]	0.88 [0.81 - 0.95]	0.90 [0.79 - 1.03]
Information & communication	0.96 [0.87 - 1.04]	0.87 [0.77 - 0.98]	1.07 [0.86 - 1.34]
Other services	1.26 [1.17 - 1.37]	1.04 [0.94 - 1.14]	1.07 [0.90 - 1.27]
Public admin, education	1.27 [1.18 - 1.37]	0.74 [0.68 - 0.82]	0.55 [0.47 - 0.65]
Real estate	1.03 [0.92 - 1.15]	0.86 [0.74 - 0.99]	1.00 [0.79 - 1.26]
Specialised activities, admin & business support	1.07 [1.00 - 1.14]	0.93 [0.86 - 1.01]	0.94 [0.82 - 1.08]
Seniority			
Employee seniority > 5 years	1.06 [1.03 - 1.1]	1.02 [0.98 - 1.07]	1.28 [1.20 - 1.36]
Administrative situation			
Adaptation of the workstation	2.73 [2.60 - 2.87]	4.17 [3.95 - 4.40]	2.87 [2.65 - 3.1]
Disabled worker	2.83 [2.58 - 3.12]	5.07 [4.61 - 5.59]	7.55 [6.72 - 8.49]
Invalidity pension	1.45 [1.22 - 1.71]	2.12 [1.80 - 2.50]	3.61 [3.01 - 4.33]
Long and/or iterative work stoppages	1.51 [1.43 - 1.59]	2.02 [1.90 - 2.14]	5.08 [4.73 - 5.46]
Long-term conditions	2.33 [2.14 - 2.54]	3.75 [3.44 - 4.10]	2.71 [2.40 - 3.06]
Permanent disability	2.02 [1.51 - 2.70]	2.85 [2.12 - 3.83]	4.45 [3.18 - 6.23]

**Table 3:** Multivariate analysis of PDRI levels according to medical characteristics.

	PDRI 1 (VS 0) OR	PDRI 2 (VS 0) OR	PDRI 3 (VS 0) OR
Disorders			
Cancers	1.82 [1.56 - 2.13]	2.27 [1.93 - 2.67]	2.18 [1.75 - 2.72]
Cardiovascular disorder	2.05 [1.75 - 2.39]	2.75 [2.35 - 3.23]	2.99 [2.43 - 3.69]
Diabetes	2.27 [2.04 - 2.54]	3.77 [3.38 - 4.22]	3.49 [2.98 - 4.08]
High blood pressure	2.27 [2.12 - 2.43]	3.90 [3.63 - 4.18]	2.83 [2.54 - 3.15]
Hypercholesterolemia	1.58 [1.40 - 1.79]	2.15 [1.89 - 2.45]	1.40 [1.13 - 1.72]
Kidney disorders	3.03 [1.65 - 5.54]	5.06 [2.77 - 9.26]	5.57 [2.63 - 11.8]
MSD arms	1.94 [1.46 - 2.58]	2.95 [2.21 - 3.94]	3.80 [2.70 - 5.34]
MSD back	1.76 [1.68 - 1.84]	2.05 [1.94 - 2.16]	2.16 [2.00 - 2.34]
MSD cervical	1.59 [1.46 - 1.73]	1.80 [1.64 - 1.98]	2.05 [1.81 - 2.32]
MSD hand	1.74 [1.50 - 2.02]	2.31 [1.98 - 2.69]	3.21 [2.67 - 3.86]
MSD lower limb	1.28 [1.16 - 1.42]	1.58 [1.42 - 1.75]	1.78 [1.55 - 2.04]
MSD shoulder	2.33 [1.99 - 2.72]	3.46 [2.96 - 4.05]	6.23 [5.23 - 7.43]
Neurological disorders	2.15 [1.99 - 2.32]	2.99 [2.75 - 3.26]	2.85 [2.51 - 3.24]
Non-localised MSD	2.59 [2.27 - 2.96]	3.99 [3.47 - 4.58]	4.25 [3.51 - 5.16]
Mental disorders	2.55 [2.38 - 2.73]	4.02 [3.75 - 4.32]	8.79 [8.10 - 9.54]
Respiratory disorders	1.78 [1.67 - 1.90]	2.62 [2.44 - 2.82]	1.87 [1.65 - 2.13]
Secondary MSD	2.91 [2.55 - 3.31]	5.40 [4.74 - 6.16]	5.74 [4.87 - 6.77]
Sleep disorders	1.94 [1.72 - 2.20]	2.64 [2.33 - 3.01]	2.86 [2.40 - 3.41]
Vision disorders	1.32 [1.27 - 1.38]	1.48 [1.42 - 1.55]	1.15 [1.06 - 1.24]
BMI (ref: BMI ≤ 25 kg/m2)			
25 < BMI ≤ 30 kg/m2	1.33 [1.29 - 1.37]	1.27 [1.22 - 1.31]	0.66 [0.62 - 0.7]
BMI > 30 kg/m2	1.51 [1.45 - 1.58]	1.50 [1.42 - 1.58]	0.82 [0.74 - 0.9]
History			
BMI formerly > 25 kg/m2 (H)	1.30 [1.11 - 1.52]	1.31 [1.09 - 1.57]	1.03 [0.77 - 1.38]
Cardiovascular disorders (H)	2.30 [1.89 - 2.81]	3.08 [2.51 - 3.78]	3.03 [2.27 - 4.04]
Diabetes (H)	0.93 [0.73 - 1.19]	0.69 [0.52 - 0.91]	0.65 [0.42 - 1.03]
High blood pressure (H)	1.37 [1.16 - 1.62]	1.30 [1.08 - 1.58]	1.05 [0.76 - 1.44]
MSD back (H)	2.20 [2.00 - 2.42]	2.15 [1.92 - 2.42]	1.46 [1.19 - 1.79]
MSD cervical (H)	2.15 [1.78 - 2.60]	2.20 [1.77 - 2.73]	2.17 [1.58 - 2.98]
MSD lower limb (H)	1.56 [1.36 - 1.80]	1.76 [1.50 - 2.07]	1.19 [0.90 - 1.57]
MSD upper limb (H)	1.95 [1.72 - 2.21]	2.35 [2.06 - 2.69]	2.32 [1.92 - 2.79]
Neurological disorders (H)	1.81 [1.54 - 2.11]	2.17 [1.83 - 2.58]	2.02 [1.56 - 2.62]
Psychological disorders (H)	2.18 [1.97 - 2.41]	2.39 [2.14 - 2.67]	2.14 [1.84 - 2.50]
Respiratory disorders (H)	1.55 [1.40 - 1.71]	1.36 [1.20 - 1.56]	1.06 [0.82 - 1.36]
Secondary MSD (H)	1.7 [1.38 - 2.09]	1.98 [1.58 - 2.49]	1.66 [1.16 - 2.37]
Sleep disorders (H)	1.57 [1.18 - 2.1]	1.88 [1.37 - 2.57]	2.01 [1.26 - 3.22]
Vision disorders (H)	1.15 [1.00 - 1.32]	1.44 [1.23 - 1.69]	1.17 [0.87 - 1.57]

**Table 4:** Multivariate analysis of PDRI levels according to professional exposure.

	PDRI 1 (VS 0) OR	PDRI 2 (VS 0) OR	PDRI 3 (VS 0) OR
Psychosocial risk factors			
Values conflicts	2.36 [2.14 - 2.62]	2.87 [2.56 - 3.2]	3.97 [3.45 - 4.57]
Emotional demands	1.34 [1.25 - 1.43]	1.46 [1.35 - 1.58]	1.25 [1.10 - 1.41]
Job insecurity	2.20 [1.93 - 2.51]	2.99 [2.59 - 3.44]	4.14 [3.47 - 4.94]
Lack of autonomy	1.12 [0.98 - 1.28]	1.39 [1.19 - 1.61]	1.79 [1.47 - 2.18]
Poor interpersonal relationships	2.76 [2.56 - 2.97]	3.88 [3.58 - 4.20]	6.05 [5.46 - 6.71]
Workload	1.69 [1.61 - 1.78]	1.80 [1.69 - 1.92]	1.95 [1.78 - 2.14]
Violence	1.02 [0.97 - 1.08]	1.08 [1.01 - 1.15]	1.11 [1.01 - 1.23]
Organisational factors			
Business trip	0.92 [0.86 - 0.98]	0.87 [0.79 - 0.95]	0.82 [0.69 - 0.97]
Shared workspace	1.04 [0.97 - 1.12]	1.19 [1.09 - 1.30]	1.03 [0.86 - 1.23]
Regular contact with the public	0.91 [0.88 - 0.94]	0.88 [0.85 - 0.92]	0.96 [0.90 - 1.03]
Frequent night shift	0.90 [0.86 - 0.95]	0.95 [0.90 - 1.01]	0.78 [0.70 - 0.87]
Solitarywork	1.07 [1.00 - 1.14]	1.20 [1.11 - 1.30]	1.17 [1.01 - 1.35]
Non-standard hours	0.93 [0.90 - 0.97]	0.94 [0.89 - 0.98]	0.82 [0.75 - 0.89]
Remote work	0.90 [0.87 - 0.94]	0.73 [0.69 - 0.77]	0.48 [0.42 - 0.54]
Shift work	1.23 [1.13 - 1.33]	0.74 [0.65 - 0.84]	0.84 [0.67 - 1.05]
Workload variability or unpredictable	1.33 [1.20 - 1.48]	1.21 [1.05 - 1.39]	1.09 [0.84 - 1.41]
Working under time constraints	1.18 [1.11 - 1.26]	0.93 [0.86 - 1.00]	0.85 [0.74 - 0.98]
Repetitiveness			
Non-localised repetitive movements	1.02 [0.99 - 1.06]	1.19 [1.14 - 1.23]	0.96 [0.90 - 1.03]
Repetitive lower limb movements	0.75 [0.54 - 1.02]	0.64 [0.40 - 1.00]	0.57 [0.24 - 1.36]
Repetitive upper limb movements	1.18 [1.12 - 1.25]	0.96 [0.89 - 1.04]	0.89 [0.78 - 1.03]
Repetitive spinal movement	0.63 [0.48 - 0.84]	0.66 [0.45 - 0.97]	0.53 [0.24 - 1.18]
Posture			
Lower limb posture	0.74 [0.66 - 0.82]	0.62 [0.54 - 0.72]	0.68 [0.52 - 0.87]
Non-localised posture	1.23 [1.19 - 1.28]	1.22 [1.17 - 1.27]	0.89 [0.83 - 0.95]
Spinal posture	0.87 [0.8 - 0.95]	0.99 [0.88 - 1.11]	0.92 [0.75 - 1.13]
Upper limb posture	1.40 [1.27 - 1.55]	1.43 [1.26 - 1.63]	1.15 [0.90 - 1.46]
Whole body posture	1.02 [0.99 - 1.05]	0.88 [0.85 - 0.92]	1.06 [0.99 - 1.13]
Physical exposure			
Bad weather	1.06 [0.94 - 1.20]	1.08 [0.93 - 1.26]	1.19 [0.96 - 1.48]
Electricity	1.07 [1.02 - 1.12]	1.05 [0.99 - 1.12]	0.86 [0.76 - 0.96]
Extreme temperatures	0.94 [0.9 - 0.98]	1.00 [0.95 - 1.06]	1.03 [0.94 - 1.13]
Fires & explosions	0.91 [0.88 - 0.95]	0.84 [0.80 - 0.88]	0.72 [0.66 - 0.79]
Ionizing radiation	0.86 [0.78 - 0.95]	0.68 [0.59 - 0.77]	0.49 [0.38 - 0.62]
Noise	1.11 [1.01 - 1.22]	0.96 [0.85 - 1.08]	1.33 [1.10 - 1.61]
Professional road risks	1.09 [1.06 - 1.12]	0.99 [0.95 - 1.03]	0.82 [0.77 - 0.88]
Risk of falls from height	0.67 [0.63 - 0.71]	0.58 [0.54 - 0.63]	0.73 [0.64 - 0.83]
Risk of falls at the same-level	1.18 [1.12 - 1.24]	1.23 [1.15 - 1.31]	1.46 [1.33 - 1.61]
Hand-arm vibration	1.01 [0.95 - 1.08]	1.07 [0.99 - 1.17]	1.01 [0.86 - 1.19]
Whole body vibration	0.68 [0.62 - 0.75]	0.60 [0.53 - 0.68]	0.64 [0.53 - 0.77]
Computer work	0.91 [0.88 - 0.94]	0.88 [0.85 - 0.92]	0.57 [0.53 - 0.61]
Heavy work	1.08 [1.04 - 1.11]	1.10 [1.06 - 1.15]	1.25 [1.17 - 1.34]
Chemical exposures			
Chemical	0.94 [0.91 - 0.97]	0.95 [0.91 - 0.99]	0.82 [0.77 - 0.88]

The ICC resulting from the application of the hierarchical model based on professional category and sector of activity is < 0.001. The variation between the ORs for each variable for each of the five subsamples does not exceed the 5% threshold. The AUC of the model applied to each subsample varies between 0.824 and 0.82. That of the overall model is 0.822.

At each iteration of λ between 0 and 100, the AUC remains at 0.822. Beyond λ = 1000, the AUC deteriorates (AUC < 0.820).

Some trends such as the over-representation of high PDRIs among white-collar workers (OR PDRI3: 1.63) observed in the univariate analysis were also confirmed in the multivariate analysis. The same situation was observed for the age category [50–61], with a PDRI2 OR of 2.95 and a PDRI3 OR of 5.17.

The different groups of disorders studied all appear to be PDR factors. Within these categories, the highest OR was for mental health conditions (8.79), followed by shoulder MSDs (6.23).

Moreover, there was some significant over-representation of high PDRIs for the different categories of medical history such as upper limb MSDs (OR PDRI2: 2.35; PDRI3: 2.32). This was also the case for employees benefiting from long or iterative work stoppage (PDRI3: 5.08).

Regarding occupational exposure, “poor interpersonal relationships” (OR PDRI1: 2.46; PDRI2: 3.88; PDRI3: 6.05) and “values conflicts” (OR PDRI1: 2.36; PDRI2: 2.87; PDRI3: 3.97) were the riskiest. Most of the “physical” occupational exposures were not statistically significant. Some physical exposure such as lower limb posture (OR PDRI3: 0.68) or whole-body vibrations (PDRI3: 0.64) appear to be ‘protective’. Exposure to noise (OR PDRI3: 1.33) and heavy work (OR PDRI3: 1.25) were associated with a PDRI3.

## Discussion

### Summary of key findings and comparison with previous literature

The main findings of our study show that advanced age, female gender, non-managerial status, MSDs, and mental disorders are risk factors for PD, consistent with the results of previous studies on risk factors for work disability or job unsuitability.

With regard to age, Berg et al. [24] reported that work capacity as assessed by the WAI decreases with increasing age, and Courtois et al. found higher rates of job unsuitability among workers over the age of 55 [9]. For women, a multicentric study showed they are 1.48 times more likely than men to be declared unfit for work, even after adjusting for age and sector [25]. The same study shows that this gap between women and men widens after the age of 40, with a difference in incidence rates of 2.5 points before the age of 40 compared to 4.5 points after the age of 40 [25]. In addition, women are two to three times more likely than men to develop disabling MSDs (e.g., carpal tunnel syndrome, tendonitis) and twice as likely to be declared unfit for work due to mental disorders [26,27]. Finally, a multicenter study [28] shows that disability due to MSDs is more common in women over 55 (OR 6.42).

For MSDs, Berg’s systematic review of the literature [24] supports our findings by objectively showing that 50% of cases of incapacity are linked to the presence of MSDs. Furthermore, the increased risk of PD in subjects with shoulder MSDs is consistent with the data from Siren et al, who found that “working” life expectancy was reduced in people with non-traumatic shoulder injuries. For example, compared to the general population, the life expectancy of workers with shoulder injuries is reduced by 8.01 years for 30-year-olds, 4.55 years for 40-year-olds, and 2.10 years for 50-year-olds [29].

For mental disorders, Courtois et al. reported in 2023 that their presence is the cause of 29% of cases of incapacity [9]. In 2017, it was found that incapacity due to mental disorders (36.8%) was associated with depressive episodes, anxiety disorders, and recurrent depressive disorders in 45.6%, 23.0%, and 13.3% of cases, respectively [30].

For occupational risk factors, our results do not show a very high risk of vulnerability for physical workload, whereas in the literature, four out of seven studies describe a decrease in WAI when it is high [24]. On the other hand, this high risk of PD exists for psychosocial risk factors such as poor workplace relationships and value conflicts, in line with the literature review by Duchaine et al. [31].

### Interpretation and potential mechanisms

In the results of our study, certain risk factors show a gradient effect, such as age, for which a worker’s vulnerability risk increases by approximately 0.7 times with each successive age group.

As for gender, the increased risk of PD observed among women could be explained by the fact that women seek medical advice for symptoms earlier than men. This phenomenon also accelerates the diagnosis of incapacity among women compared to men, who often delay seeking medical advice. Furthermore, women have lower average muscle strength (approximately 60–70% of that of men), which exposes them to more repetitive microtrauma, and they have anatomical differences (e.g., narrower shoulders, thinner wrists, etc.) that lead to increased stress on their joints. More often than men, they combine professional work with domestic tasks and work in emotionally demanding sectors (social work, care, etc.) exposing them to stress and violence.

Other factors such as MSDs or mental health conditions represent a significant baseline risk coupled with a “leverage effect.” Thus, for MSDs as a whole, the risk of vulnerability, which is found to be higher overall, increases further for shoulder locations when classifying employees as PDRI3. Thus, the presence of an MSD in the shoulder induces an excess risk of moving to PDRI3 with an OR of 6.06, whereas for the cervical spine this risk is 1.87.

This result could be explained by the fact that MSDs located in the shoulder are often intense and long-lasting, with limited effect from pain relief treatments, making it more difficult to remain in the job. According to Chu et al [32], a complete rupture of the supraspinatus tendon increases the risk of losing one’s job by more than eightfold.

For mental disorders, the leverage effect is even greater, with an OR of more than 8 for PDRI3 classification. This high OR is probably linked to a type of psychiatric disorder or its intensity, which could not be specified in our study. The plurality of diagnoses falling into this category may lead to variability in the data entered by healthcare professionals, who do not always have all the information necessary for an accurate diagnosis. In addition, the repercussions of the same diagnosis differ from one worker to another.

According to Johnston [33], in the case of depressive disorders, the presence or severity of certain symptoms is correlated with increased absenteeism and decreased work performance.

For some variables, the OR is higher for PDRI2 than for PDRI3, whereas one would expect to see an OR that is at least as high for PDRI3. This mainly concerns long-term conditions, workplace accommodations, and high blood pressure. This phenomenon could be explained by the fact that these factors are not of a nature or intensity sufficient to induce very high employment vulnerability, or that they lead employees to leave their jobs as soon as the severity of the condition increases, contributing to a selection bias. As our study was conducted on a population of active workers, data on employees who were laid off or resigned are not taken into account.

This selection bias may explain this phenomenon, which has also been observed among employees exposed to repetitive movements or vibrations of the upper limbs. Workers with a condition related to these exposures may have left their jobs, benefited from adjustments or been redeployed when they reached the highest level of vulnerability.

The reduction in risk associated with seniority in the workplace could be linked to a “healthy worker” effect. However, for obesity, which is associated with an increased risk of chronic diseases, a meta-analysis [34] revealed that obese workers were 20% more likely to remain in the same job for more than 10 years compared to non-obese employees. This stability mainly concerns sedentary jobs or manual occupations that are not physically demanding, in which obesity is overrepresented [35]. The more pronounced difference in favor of a decrease in job turnover among obese individuals compared to those who are not obese can also be explained by the fact that this morbidity is more common after the age of 40, an age at which seniority in the job is naturally higher [36].

### Practical and political implications

The PDRI appears to be a useful tool for occupational health professionals to identify vulnerable employees at an early stage, whether at a collective or individual level.

At the collective level, the classification of several employees of the same company in the vulnerable category according to one or more identified occupational risk factors could lead the occupational health team to provide the employer with prevention advice targeted at the risk factor.

In the case of a chronic condition, a program could be established by the occupational health team in conjunction with the employer to prevent the assignment of the individuals concerned to physically demanding jobs. For fairly common conditions such as obesity, hypertension, and cervical MSDs, this program would aim to break the cycle of seniority in a sedentary position by promoting, for example, alternating with a position requiring mobility.

At the individual level, identifying factors that increase the risk of PDRI should enable occupational health professionals to intervene as early as possible in the prevention process. The identification of weak signals in the workplace—such as persistent fatigue, subclinical musculoskeletal discomfort, or emerging symptoms of stress—could lead to recommendations for adjustments/modifications to the workstation or even training for a new assignment, and to at least a temporary increase in the frequency of health monitoring for a worker, thereby possibly preventing subsequent sick leave.

Whether collective or individual, by improving workers’ quality of life at work, these various preventive measures could enable companies to improve worker efficiency and reduce the costs of absenteeism.

Thus, integrating the PDRI screening tool into occupational health monitoring programs could promote the implementation of appropriate interventions to maintain employability. This approach is also consistent with national strategies aimed at helping individuals stay healthy at work and keep their jobs.

### Statistical considerations

In view of the methodological considerations and the results of the statistical tests (ICC < 0.001), the authors decided not to retain the results of the hierarchical model. Nevertheless, if it turned out that the observations were not independent, then the standard errors would be underestimated, artificially increasing the statistical significance of the results. Furthermore, we can consider that while the risk of PD is multifactorial, it is nonetheless independent of context.

The stability of ORs between subsamples and the stability of AUC when applying the regression model to each sample rule out the hypothesis of overfitting by the model. Nevertheless, methodologically, the PDRI includes the presence of pathology, which is also integrated into the independent variables, in its assessment criteria for practitioners. It is conceivable that the analytical model cannot be perfectly generalized in a predictive framework. It should nevertheless be noted that the PDRI is not limited to documenting a pathology; it takes into account the way in which health interacts with work. A future study is planned to test the generalizability of the model. Pathologies are documented before the PDRI is assessed, and the idea is to seek a predictive model for the PDRI even before the professional has time to assess it.

The stability of the results and performance of the model across the five subsamples rules out the hypothesis that the associations are significant solely because of the large sample size. Nevertheless, for ORs closest to 1, the interpretation as a risk factor for disengagement should be qualified.

### Strengths and limitations

This study was based on data collected in daily practice by occupational health professionals, with a multifactorial view of PD. A global approach was adopted taking into account the health of the worker, their disorders, and their medical history. Additionally, the work and various occupational constraints were studied according to the sector of activity, posture, and work organisation. The worker’s feelings were also considered with the assessment of their well-being at work and psychosocial risk factors.

The PDRI is a relatively recent score. Its external validity and inter-judge validity has been validated but questions remain regarding the heterogeneity for PDRI level 2 [12].

The precision level of data coding, such as further information about the kind of exposure, can vary according to the health professional due in particular to the documentation available on companies’ activities. Moreover, if numerous occupational exposures are reported, the same exposure code may be entered even though the “intensity” is not identical between two workers. This could partly explain the fact that some “exposure” variables, or even protective factors, appear to be insignificant. For example, it might be thought that computer input coded as “repetitive movement of MS” will not have the same effect as work on an assembly line, which is also coded in the “repetitive movement of MS” category.

Some variables collected during the visit were not quantified using validated questionnaires. This was particularly the case for habits and lifestyles. To assess nutrition, for example, the possible answers during the visit were “unbalanced - not very balanced - fairly balanced - balanced”. This grading scale is based on the information provided by the employee and their assessment by the healthcare professional that may vary in the degree of precision given. Furthermore, the assessment may also vary from one healthcare professional to another. This was also the case for the assessment of sleep quality (regular sleep problems – occasional – no problem) or physical activity (none-occasional-regular-intensive). However, some habits can be assessed using validated questionnaires, as is the case for smoking or alcohol consumption, for example, but these are rarely used in practice by healthcare professionals.

## Conclusion

In this study, clinical factors such as mental and musculoskeletal disorders and professional factors such as poor working relationships, job insecurity and values conflicts appear to be risk factors for professional disengagement risk. These results reinforce the importance of targeted prevention on avoidable risk factors. Future research should refine the accuracy of the variables, better distinguish gradients and thresholds of risk, and validate the use of standardized tools for lifestyle habits.

## Supporting information

S1 Table WDRI evaluation grid. (DOCX)

S2 Table Disorders, medical history and exposures codes. (DOCX)

S1 Fig Comparison of WDRI 0–1 VS WDRI 2–3 results. (DOCX)
