# Supplementary material for: Modelling the risk of professional disengagement from a cohort study of 181,676 workers in the south of France
Source: PLoS One. 2026 Mar 31;21(3):e0346149. doi: 10.1371/journal.pone.0346149 (PMC13037995; doi:10.1371/journal.pone.0346149)
Supplement: S1 Table — (DOCX) [file pone.0346149.s001.docx]

**S1 Table. WDRI evaluation grid**

| **SOMATIC OR MENTAL DISORDERS**  **criteria** | **Level of**  **WDRI** | **WELL-BEING**  **AT WORK (17,18)**  **criteria** |
| --- | --- | --- |
| **NO** disorder identified | **0**  No health-work impact  **Zero risk** | Well-being score at work ≥ 7 |
| **Risk factor(s)** known to cause life-altering diseases  OR **Medical history** likely to reactivate | **1**  Possible health-work impact in the long term  **Low risk** | **Well-being score at work < 7**  **NO** health impact  **NO** questioning of future in the company |
| **Current Disease(s)**  OR **Complaint(s)** of the worker  OR **Clinical sign(s)** identified by physician | **2**  Possible health-work impact  in the medium term  **Medium Risk** | **Well-being score at work < 7**  AND **Health impact**  OR **No longer sees a sustainable future in the company** |
| **WDRI level 2**  AND **Work discomfort**  expressed by the worker  or observed by physician | **3**  Proven health-work impact  **High Risk** | **Well-being score at work < 7**  AND **Health impact**  AND **No longer sees a sustainable future in the company** |
