# Supplementary material for: Modelling the risk of professional disengagement from a cohort study of 181,676 workers in the south of France
Source: PLoS One. 2026 Mar 31;21(3):e0346149. doi: 10.1371/journal.pone.0346149 (PMC13037995; doi:10.1371/journal.pone.0346149)
Supplement: S2 Table — (DOCX) [file pone.0346149.s002.docx]

**S2 Table. Disorders and medical history (ICD10 and ICD11 coding)**

| **Disorders/History** | **ICD10 codes** | **ICD11 codes** |
| --- | --- | --- |
| MSD arm/elbow | G56, G562, G563, G569, M752, M771, M772, S440, S46, S461, S462, S463, S468, S469, S540 | 8C101, 8C102, 8C10Z, FB40Y, FB551, FB552, NC140, NC161Z, NC162Z, NC163Z, NC16Z, NC340 |
| MSD shoulder | M75, M750, M751, M753, M754, M758, M759, S460 | FB403, FB530, FB531, FB532, FB53Z, NC160Z |
| MSD hand/wrist | G560, G561, I730, M18, M653, M654, M720, M931, S60, S607, S608, S609, S633, S64, S640, S641, S642, S643, S644, S647, S648, S65, S651, S655, S66, S660, S661, S662, S663, S664, S665, S667, S668, S669, S69 | 8C100, 8C10Y, BD42Z, FA02Z, FB404, FB405, FB510, FB810, NC51, NC512, NC544Z, NC550, NC551, NC552, NC553, NC55Y, NC55Z, NC561Z, NC565Z, NC56Z, NC570Z, NC571Z, NC572Z, NC573Z, NC574Z, NC575Z, NC577, NC57Z, NC5Y |
| MSD cervical | M43, M50, M436, M530, M531, M542, M852, M953 | FA71, FA80, FA71, FB803, FC001, ME840 |
| MSD back | M43, M47, M314, M40, M400, M41, M411, M412, M413, M414, M415, M418, M419, M438, M48, M51, M53, M532, M533, M538, M539, M54, M541, M543, M544, M545, M546, M548, M549, M940, M954, M955, M992, M993, M994, M995, M997 | 4A441, 8B93Z, FA700, FA701, FA70Z, FA72Z, FA803, FB0Y, FB10, FB1Z, FB82Z, FC002, FC003, ME841, ME842, ME843, ME84Z, ME931, ME932, ME933, ME934, ME936, 8B42, FA81, FA84, FA8Z, FB1Z |
| MSD lower limbs | M16, M17, M200, M201, M202, M203, M204, M214, M216, M22, M23, M303, M712, M722, M76, M773, M774, M775, M930 | 4A445, FA00Z, FA01Z, FA300, FA301, FA302, FA30Y, FA315, FA31Z, FA32Z, FA33Z, FB502, FB51Y, FB543, FB544, FB54Z, FB822 |
| Secondary MSD | M00, M02, M05, M08, M10, M14, M34, M35, M42, M45, M60, M67, M72, M80, M82, M85, M93, M000, M012, M015, M052, M053, M059, M060, M061, M064, M069, M07, M070, M073, M074, M080, M081, M083, M089, M09, M090, M100, M103, M109, M112, M119, M122, M13, M130, M138, M139, M140, M252, M301, M313, M316, M32, M321, M328, M329, M330, M331, M332, M341, M348, M349, M350, M352, M353, M354, M358, M359, M362, M410, M420, M46, M461, M469, M49, M495, M608, M609, M726, M790, M797, M800, M802, M804, M81, M810, M812, M818, M819, M840, M841, M842, M844, M859, M86, M861, M871, M872, M878, M879, M88, M888, M889, M892, M896, M901, M91, M911, M919, M924, M925, M926, M929, M932, M941, M960, M966 | 1B71, 4A42, 4A43, 4A44, 4A4Z, 4A62, FA11, FA1Z, FA20, FA22, FA24, FA25, FA27, FA38, FA85, FA92, FA9Z, FB32, FB3Z, FB42, FB51, FB80, FB81, FB82, FB83, LD28, 1B71Z, 1C1G1, 1E1Z, 4A400, 4A410, 4A411, 4A422, 4A42Z, 4A434, 4A43Z, 4A442, 4A44A, 4A44Y, 4A4Z, 4A62, FA11Y, FA1Z, FA20Z, FA21Y, FA21Z, FA22, FA23, FA241, FA242, FA24Z, FA250, FA251, FA252, FA262, FA26Z, FA271, FA2Z, FA37Y, FA383, FA701, FA920, FA9Z, FB0Y, FB0Z, FB32, FB3Z, FB56, FB807, FB808, FB809, FB80B, FB80Z, FB812, FB813, FB81Z, FB821, FB823, FB831, FB84Z, FB85Z, FB86Z, FB8Y, FC010, FC016, MG300 |

**FB50Z,* *FB501 bursitis excluded from MSD*

**S2 Table. Disorders and medical history (ICD10 and ICD11 coding) (continuation)**

| **Disorders/History** | **ICD10 codes** | **ICD11 codes** |
| --- | --- | --- |
| Mental health disorders | F05, F06, F07, F09, F10, F11, F12, F13, F14, F15, F16, F17, F19, F20, F21, F22, F23, F25, F28, F29, F30, F31, F32, F33, F34, F38, F39, F40, F41, F42, F43, F44, F45, F48, F50, F99 | 6A20, 6A21, 6A22, 6A23, 6A24, 6A2Z, 6A4Z, 6A60, 6A62, 6A6Z, 6A70, 6A71, 6A72, 6A73, 6A7Z, 6A8Z, 6B00, 6B01, 6B02, 6B03, 6B04, 6B0Z, 6B20, 6B23, 6B40, 6B43, 6B4Z, 6B60, 6B6Z, 6B80, 6B81, 6B8Z, 6C20, 6C40, 6C41, 6C43, 6C44, 6C45, 6C47, 6C48, 6C49, 6C4A, 6C4Z, 6C9Z, 6D70, 6D71, 6D72, 6E0Z, 6E61, 6E62, 6E63, 6E68, 6E6Z, 6E8Z, QE84 |
| Cancers | 30, C31, C32, C34, C37, C38, C39, C40, C41, C50, C71, C81, C82, C83, C84, C85, C86, C88, C90, C91, C92, C94, C95, C96, H40 | 2A00, 2A20, 2A44, 2A60, 2A80, 2A81, 2A82, 2A83, 2A84, 2A85, 2A86, 2A8Z, 2A90, 2B01, 2B0Z, 2B30, 2B31, 2B33, 2B3Z, 2B5Z, 2C21, 2C22, 2C23, 2C25, 2C26, 2C27, 2C29, 2C6Z, 9C60, 9C61, 9C6Z |
| Cardiovascular disorders | I05, I06, I07, I08, I09, I11, I12, I15, I21, I24, I26, I27, I28, I31, I34, I35, I42, I49, I50, I51, I63, I64, I69 | 8B11, 8B20, 8B25, BA01, BA02, BA04, BA41, BA43, BA4Z, BB00, BB01, BB02, BB0Z, BB22, BB24, BB25, BB2Z, BB60, BB61, BB62, BB6Z, BB70, BB71, BB72, BB7Z, BB81, BC00, BC0Z, BC20, BC42, BC43, BC45, BC46, BC4Z, BC60, BC70, BC71, BC80, BD10, BD11, BD1Z, BE2Y |
| Hypertension | I10 | BA00 |
| Neurological disorders | G20, G25, G35, G36, G37, G40, G43, G45, G50, G51, G52, G70, G95, G98, I60, I61, I62, I63, I64, I65, I66, I67, I69, G610 | 8A00, 8A04, 8A06, 8A0Z, 8A40, 8A41, 8A43, 8A4Z, 8A60, 8A61, 8A68, 8A6Z, 8A80, 8A85, 8B00, 8B01, 8B02, 8B0Z, 8B10, 8B11, 8B20, 8B22, 8B25, 8B2Z, 8B42, 8B82, 8B86, 8B88, 8B8Z, 8C60, 8C6Y, 8C6Z, 8D66, 8E7Z, BD55, MB21 |
| Vision disorders | H50, H52, H53, H54, H57 | 9C80, 9C8Y, 9D00, 9D01, 9D0Z, 9D42, 9D44, 9D46, 9D5Z, 9D7Z, 9D90, 9E1Z, LA11, MC18, QA00 |
| Respiratory disorders | J20, J44, J45, J93 | CA22, CA23, CA42, CB21 |
| Sleep disorders | G47, G473 | 7A4Z, 7B2Z |
| Diabetes | E10, E11, E14 | 5A10, 5A11, 5A14 |
| Hypercholesterolemia | E780, E783 | 5C800, 5C801 |
| Kidney disorders | N17, N18 | GB60, GB61, GB6Z |
| Obesity | E66, E660, E662, E668, E669 | 5B810, 5B81Z, 7A420 |

**S2 Table. Organisational factors**

| **Organisational factors** | **Anses codes** |
| --- | --- |
| Remote work | MB04, MB04ZZ |
| Variability or unpredictability workload | GA04ABAF, MA04AD, MA04AK |
| Working under time constraints | GA04ABAJ, MA03, MA03AC, MA03AF, MA03AJ, MA04AJ |
| Frequent night shift | GA01ABAB, GA01ABZZ, MA01AB, MA01ABAB, MA01ABZZ |
| Isolated work | GA04AB, GA04ABAD, MA04AH |
| Shift work | GA01AA, GA01AAAA, GA01AAAB, GA01AAZZ, MA01AA, MA01AAAA, MA01AAAB, MA01AAZZ |
| Non-standard hours | GA01AD, GA01ADZZ, MA01, MA01AD, MA01ADAB, MA01ADAC |
| Business trip | GA02, GA02AA, GA02AB, GA02AC, GA02ZZ, MA02, MA02AA, MA02AB, MA02AC, MA02ZZ |
| Co-working | MA04AF |
| Regular contact with the public | GA04ABAC, MA05AB |

**S2 Table. Biomechanical exposures**

| **Biomechanical exposures** | **Anses codes** |
| --- | --- |
| Heavy work | FA, FA01, FA02, FA02AA, FA02AAAC, FA02AAAD, FA02AAZ1, FA02AB, FA02ABAA, FA02ABAD, FA02ABZ1, FA02AC, FA02ZZ, FA06, FA06AA, FA06AB, FA06ZZ |
| Repetitive motion upper limb | FB02, FB02ZZ, FB02AG, FB02AGAA, FB02AGAB, FB02AGZZ, FB02AH, FB02AB, FB02AD |
| Repetitive movement of the spine | FB04, FB07, FB07AA, FB07AB, FB07ZZ |
| Repetitive motion lower limb | FB01, FB01AA, FB01AB, FB01ZZ |
| Non-localised repetitive motion | FB, FBZZ |
| Upper limb posture | FC03AA, FC03AAAK, FC03AAAL, FC03AAAM, FC03AAAN, FC03AAZZ, FC03, FC03ZZ, FC03AC, FC03ACAB, FC03ACZZ, FC03AD, FC03ADAA, FC03ADAB, FC03ADZZ |
| Spinal posture | FC08, FC08AA, FC08AB, FC08AC, FC08AD, FC08ZZ, FC05, FC05AD, FC05AF, FC05AG, FC05AH, FC05ZZ |
| Lower limb posture | FC02, FC02AB, FC02AC, FC02ACAB, FC02ACZZ, FC02ZZ |
| Whole body posture | FC01, FC01ZZ, FC01AB, FC01ABAC, FC01ABAD, FC01ABAF, FC01ABAG, FC01ABZZ, FC01AD, FC01ADAA, FC01ADAB, FC01ADZZ, FC01AA |
| Non-localised posture | FC, FCZZ |

**S2 Table. Physical exposures**

| **Physical exposures** | **Anses codes** |
| --- | --- |
| Extreme temperatures | DA02AA, DA02AAAA, DA02AAAB, DA02ADAA, DM01AA, DM01AAAB, DM01AAZZ, DM01ZZ, DA02AB, DA02ABAA, DA02ABAA01, DA02ABAA02, DA02ABAB, DA02ABAB01, DA02ABAB02, DA02ADAB, DM01AB, DM01ABAA, DM01ABAA01, DM01ABAA02, DM01ABAB, DM01ABAB01, DM01ABAB02, DM, DM01, DM01ABZZ, DM01AC |
| Vibration whole body | DH04, DH04AA, DR01, DR01AA, DR01AB, DR01AC, DR01ZZ |
| Vibration upper limb | DH01, DH01AA, DR02, DR02AA, DR02AB, DR02ZZ |
| Noise | DH03AAAA02, DH03AAAA03, DH03AAAB, DH03AAAB01, DS01AB |
| Ionizing radiation | DG03, DG03AB, DG03AG, DQ, DQ01, DQ02, DQ03, DQ05 |
| Bad weather | DA01 |
| Electricity | DC, DC01, DC02, DC02AB, DC02AC, DC03, DC03AC, DC05, DC06, DC07, DCZ1, DCZZ |
| Fires & explosions | DD, DD01, DD02, DDZ1, DDZZ |
| Risk of same-level falls | DJ, JM05, JM05AA, JM05AB, JM05AC, JM05AD, JM05AF, JM05AH, JM05AK, LA04AB |
| Risk of fall from one level to another | DL, LA02AB |
| Professional road risks | KB06, KB06AA, KB06AAAA, KB06AAAA01, KB06AAAA02, KB06AAAA03, KB06AAAA04, KB06AAAA05, KB06AAAA06, KB06AAAA07, KB06AAAA08, KB06AAAA09, KB06AAAAZZ, KB06AAAB, KB06AAAB01, KB06AAAB02, KB06AAAB03, KB06AAAB04, KB06AAAB05, KB06AAAB06, KB06AAAB07, KB06AAABZZ, KB06AAAC, KB06AAAC01, KB06AAAC02, KB06AAAC03, KB06AAAC04, KB06AAAC05, KB06AAACZZ, KB06ABAA01, KB06ADAB, KB06ADAC, KB06ZZ |
| Working with display screen equipment | KA01, KA03, KA03AA, KA03AB, KA03AC |

**S2 Table. Psycho-Social Risk (PSR) Factors**

| **PSR Factors** | **Anses codes** |
| --- | --- |
| Emotional demands | MA04AC, MA05, MA05AA, MA05AC, MA05AD, MA05ZZ, GA04AB, GA04ABAA, GA04ABAB, GA04ABAG, GA04ABAH, GA04ABZZ |
| Lack of autonomy | GB04, MB03AC, GB03, GB03AB |
| Damaged relations at work | GC01, GC01AB, GC01ABAA, MC01, MC01AA, MC01AAAA, MC01AAAB, MC01AAAC, MC01AAZZ, MC01AB, MC01ABAA, MC01ABAB, MC01ZZ, GC01AAAA, GC01AAAC, MC01AC, MC01ACAA, MC01ACAB, MC01ACAC, MC01ACAD, MC01ACZZ |
| Conflicting values | MA04AL, MA04AM, MC01AAAF, MD, GB08AA, GB08AB, GB08AG |
| Workload | GB02, GB02AA, GB02AAAA, GB02AAAB, GB02ZZ, MB06, MB06AA, MB06AB, MB06ZZ, MA04, MA04AA, MA04ZZ |
| Insecurity | GG05AC, MF03AB, MF03ABZZ, MF03ABAA |
| Violence | GC, GC02AA, GC02AAAA, GC02AAAB, GC02AB, MC, MC02, MC02AA, MC02AAAA, MC02AAAB, MC02AAZZ, MC02AB, MC02ABAA, MC02ABAB, MC02ABZZ, MC02AC, MC02ACAA, MC02ACAB, MC02ACZZ, MC02AD, MC02ADAA, MC02ADAB, MC02ADZZ, MC02AF, MC02AG, MC02AH, MC02ZZ, MC03, MC03AA, MC03AB, MC03ABAA, MC03ABAB, MC03ABZZ, MC03AC, MC03ACAA, MC03ACAB, MC03ACZZ, MC03AD, MC03ADAB, MC03AF, MC03AFAB, MC03AG, MC03AJ, MC03ZZ |
