# Supplementary material for: Modelling the risk of professional disengagement from a cohort study of 181,676 workers in the south of France
Source: PLoS One. 2026 Mar 31;21(3):e0346149. doi: 10.1371/journal.pone.0346149 (PMC13037995; doi:10.1371/journal.pone.0346149)
Supplement: S1 Fig — (DOCX) [file pone.0346149.s003.docx]

**S3 Figure. Comparison of WDRI 0-1 VS WDRI 2-3 for socio-professional characteristics and administrative situations**

**
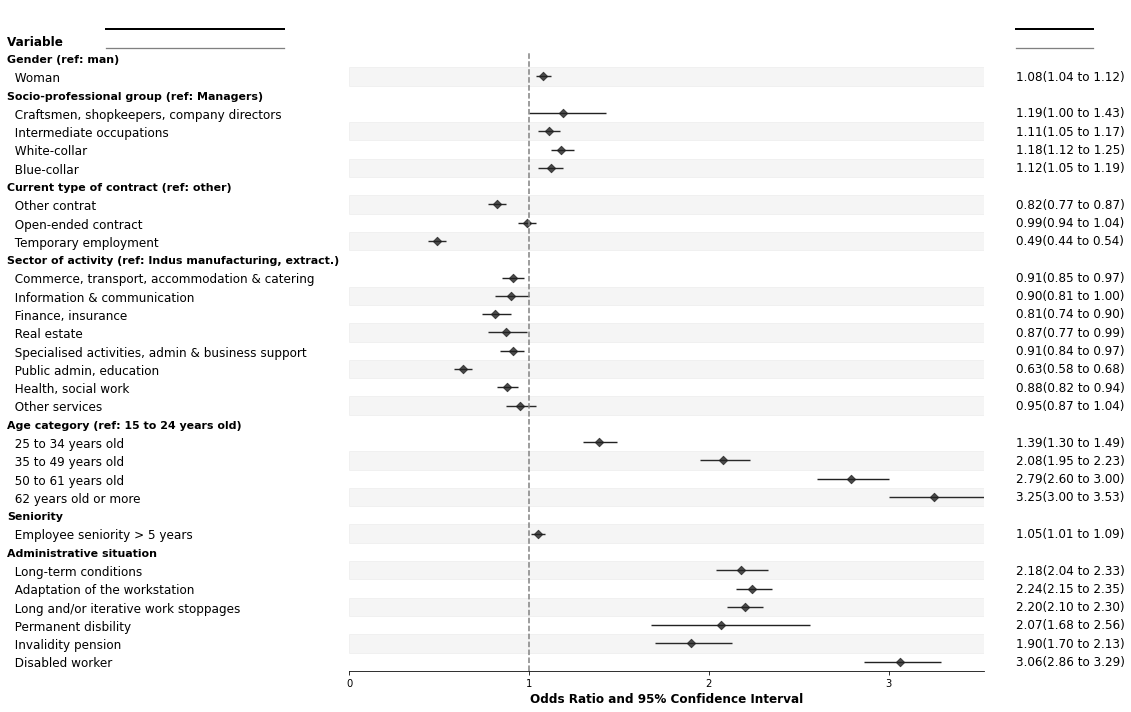
**

**S3 Figure. Comparison of WDRI 0-1 VS WDRI 2-3 for medical characteristics**

**
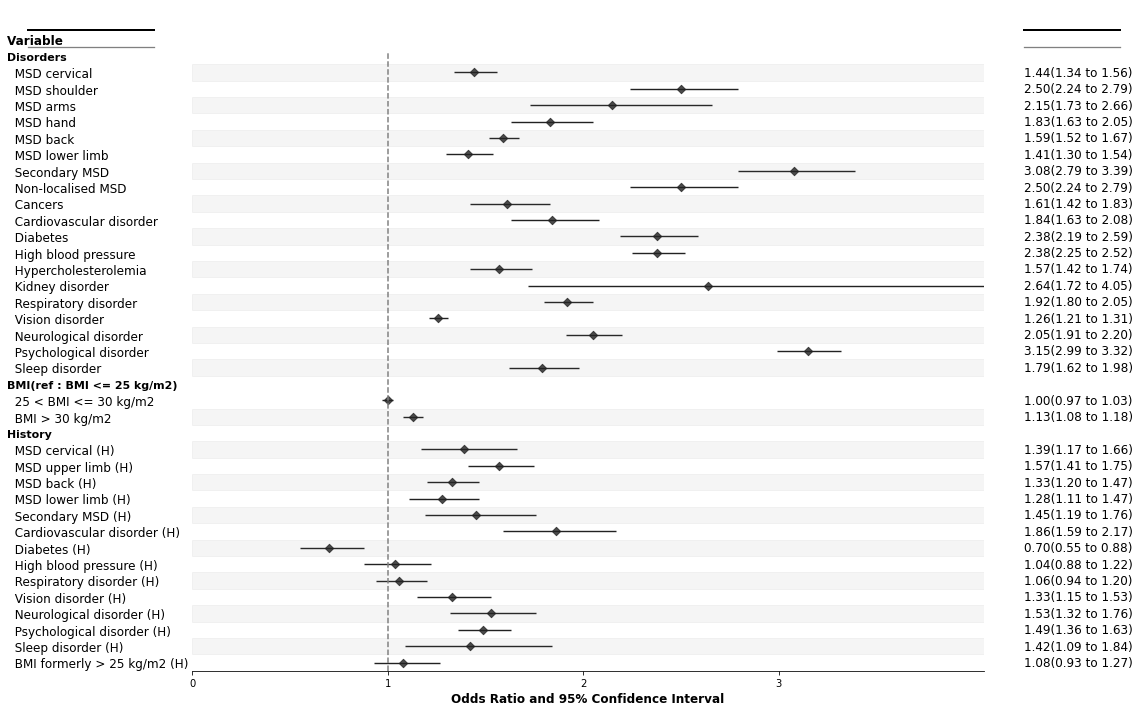
**

**S3 Figure. Comparison of WDRI 0-1 VS WDRI 2-3 for professional exposure**

**
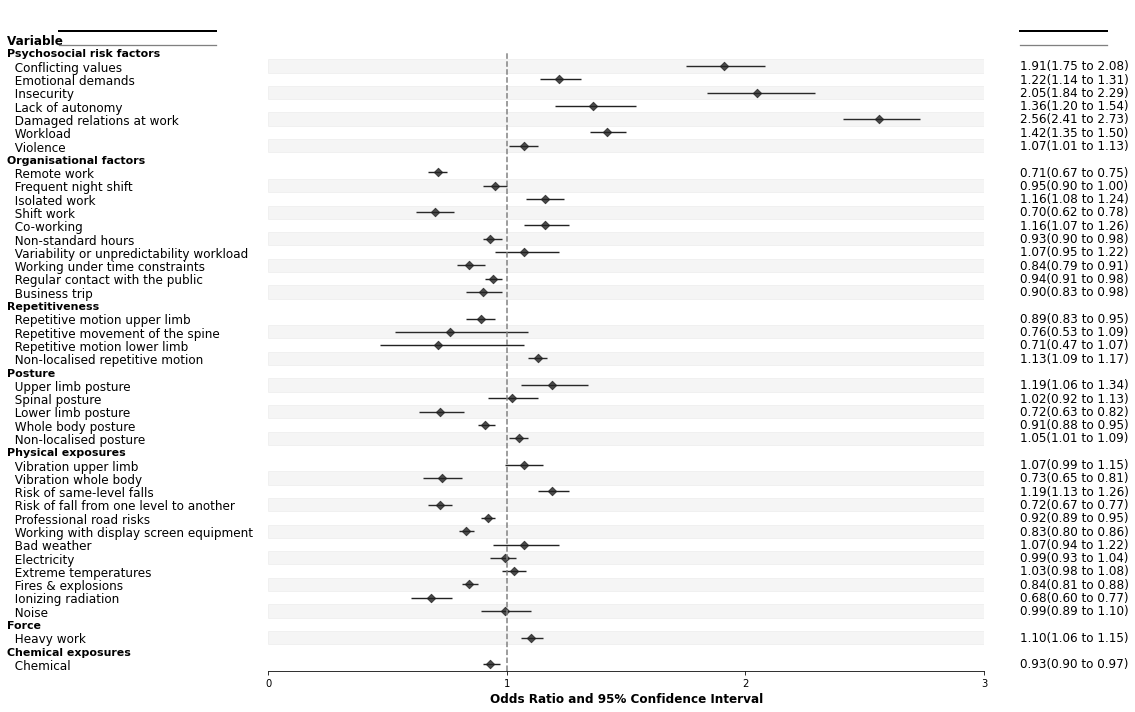
**
